# Expression profile of cuticular genes of silkworm, *Bombyx mori*

**DOI:** 10.1186/1471-2164-11-173

**Published:** 2010-03-15

**Authors:** Jiubo Liang, Liang Zhang, Zhonghuai Xiang, Ningjia He

**Affiliations:** 1 The Key Sericultural Laboratory of Agricultural Ministry, College of Biotechnology, Southwest University, Beibei, Chongqing 400715, China; 2 National Engineering Center for Beijing Biochip Technology, Life Science Parkway, Changping District, Beijing 102206, China

## Abstract

**Background:**

Insect cuticle plays essential roles in many physiological functions. During molting and metamorphosis tremendous changes occur in silkworm cuticle where multiple proteins exist and genes encoding them constitute about 1.5% of all *Bombyx mori *genes.

**Results:**

In an effort to determine their expression profiles, a microarray-based investigation was carried out using mRNA collected from larvae to pupae. The results showed that a total of 6676 genes involved in various functions and physiological pathways were activated. The vast majority (93%) of cuticular protein genes were expressed in selected stages with varying expression patterns. There was no correlation between expression patterns and the presence of conserved motifs. Twenty-six RR genes distributed in chromosome 22 were co-expressed at the larval and wandering stages. The 2 kb upstream regions of these genes were further analyzed and three putative elements were identified.

**Conclusions:**

Data from the present study provide, for the first time, a comprehensive expression profile of genes in silkworm epidermal tissues and evidence that putative elements exist to allow massive production of mRNAs from specific cuticular protein genes.

## Background

Silkworm, a model for Lepidoptera, is a holometabolous insect whose developmental stages include egg, five larval instars, pupa, and adult. During molting and metamorphosis, conspicuous and relatively abrupt changes are seen in its cuticle. Insect cuticle is mainly composed of chitin nanofibres embedded in a matrix of cuticular proteins. In procuticle, a grouping of what has been called the exo- and endocuticle, cuticular proteins bound to chitin and cross-linked with the sclerotizing agents form one of the most infrangible known biological coverings [1]. Generally, cuticle plays essential roles in many physiological functions to protect the insect's body from dehydration, the invasion of pathogens, the penetration of insecticides, and physical injury [2-5].

As an important component of cuticle, hundreds of cuticular protein sequences have been identified in over 20 species of insects [6]. Many conserved motifs were identified in this data including R&R Consensus [7], CPF&CPFL [8], Tweedle [9], and others. Among them, the cuticular protein sequences containing R&R Consensus (CPR) were extensively studied in *Anopheles gambiae*, *Drosophila melanogaster*, *Bombyx mori*, and *Apis mellifera *by the annotation of genomic data [10-13]. Togawa and coworkers subsequently examined the expression profile of 156 CPR genes in *A. gambiae *by real-time RT-PCR and found that most of them were expressed at single or multiple periods associated with molting [14].

Our bioinformatic analysis and previous work of others have identified more than two hundred cuticular protein genes in the silkworm genome [12], indicating that the silkworm employs more than 1.5% of its estimated protein-coding genes to encode cuticular proteins. These observations led us to focus on the following three questions: 1) How many genes including cuticular protein genes are expressed in silkworm epidermal tissues? 2) Is the expression of a special set of cuticular protein genes metamorphic stage-specific? and 3) Are cuticular protein genes coordinately regulated? The sequencing of the silkworm genome along with microarray technology offered us an opportunity to investigate gene expression profiles on a large scale to answer these questions. Eleven developmental stages were selected, which ranged from day 4 of the fourth instar larva to day 8 of pupa, and microarray-based expression profile analysis of all detectable genes in silkworm epidermal tissues was performed. Our data showed that a total of 6676 genes including the vast majority of silkworm cuticular protein genes were activated in selected stages, with no correlation between expression patterns and the presence of conserved motifs. In addition, twenty-six CPR protein genes distributed on chromosome 22 were co-expressed in larval and wandering stages and three common elements were identified in the 2 kb upstream region of these co-expressed CPR genes.

## Results

### Developmental expression profile of genes in epidermal tissues

**Figure 1:**
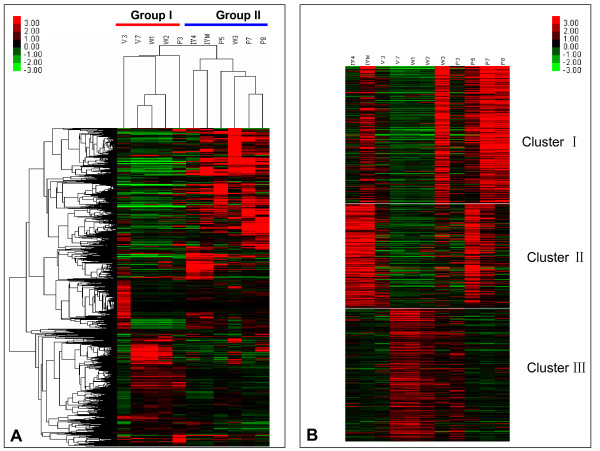
**Heat map of hierarchical clustering of 6676 genes expressed in epidermal tissues**. Genes expressed with signal intensity more than 800 were included in this analysis. Clustering was done using Cluster 3 software (clustering type: hierarchical clustering, Distance metric: Pearson correlation). The colors in the map display the relative values of all tiles within the given 11 developmental stages; green indicates the lowest expression, black indicates the intermediate expression, and red indicates the highest expression. The numerical values give the actual values on a log 2 scale, which were associated with each color. The color scale bar is shown at the top right corner of the figure. **A**: Genes clustered by the given 11 developmental stages. **B**: Genes grouped into three clusters on the basis of the similarity of expression.

In silkworm, oligonucleotide microarrays were employed to examine gene expression profiles of ten tissues on day 3 of the fifth instar larval stage, as reported by Xia *et al*. [15]. The microarray contained 22,987 70-mer oligonucleotides covering all predicted genes derived from the first draft silkworm genome sequence database [16]. Thereafter, the silkworm genomic database was updated and information refreshed [17,18], and one hundred and forty-seven additional oligonucleotide probes were designed for previously unpredicted genes. The current oligonucleotide microarray contained 23,134 probes, and the complete set of raw and normalized data from this study has been deposited in the Gene Expression Omnibus (GEO) repository (accession number GSE18878). We investigated the developmental expression profile of genes in epidermal tissues using the updated microarrays. A total of 6676 (28.9%) genes were transcribed in at least one selected stage in which clean cuticle tissues were easily isolated. As shown in Figure 1, the clustering analysis was carried out to analyze the expression profile. From the heat map of hierarchical clustering (Figure 1A), we found that the selected 11 stages were clustered into two groups. Group I contained five stages including V3, V7, W1, W2, and P3. The remaining six stages, IV4, IVM, W3, P5, P7, and P8 fell into group II. Stages V7, W1, and W2 of group I were closely clustered into one subgroup, indicating that genes shared a similar expression profile in epidermal tissues in these stages. By contrast, stages V3 and P3 were separated, indicating they had different gene expression profiles from one another which were distinct from the other in group I stages. Unlike group I, group II was further divided into two subgroups, one consisting of stages of IV4 and IVM and the other a tight cluster of stages W3, P5, P7, and P8.

To determine the significance of the expression profiles, K-means clustering was performed (Figure 1B), resulting in 6676 genes divided into three clusters. Clusters I and III comprised 2450 genes and 2373 genes, respectively. Cluster II, the smaller one, contained 1853 genes. It is worthwhile to mention that genes in the three clusters showed distinctly different expression patterns. Genes in cluster I were highly expressed at stages of W3, P7, and P8, whereas genes in cluster II were highly expressed at IV4, IVM, and P5. Genes in both clusters I and II showed low expression level at stages of V7, W1, and W2. In contrast, genes in cluster III had higher expression levels at those stages.

**Figure 2:**
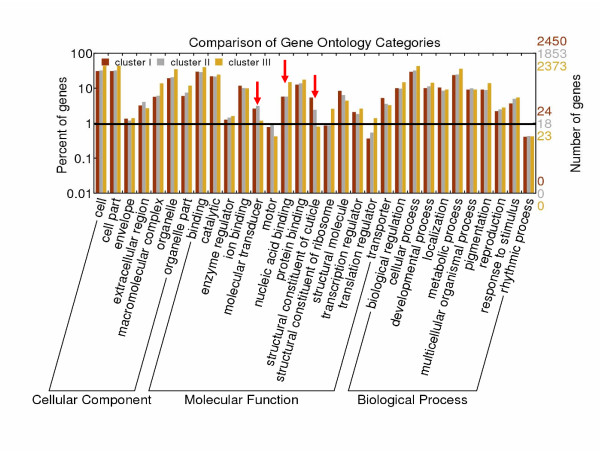
**Assignment of Gene Ontology (GO) categories of cluster I to cluster III**. This analysis was carried out using the BGI WEGO homepage mirror (GO Archive: 2008-10-01). Red arrow heads indicate the differences among the three clusters. Stages are as described in abbreviation.

Diverse expression patterns indicated genes of these three clusters might be involved in distinct functions and physiological pathways. Therefore, Gene Ontology (GO) annotations were determined for genes in the three clusters (Figure 2). As shown in the GO map, genes in all clusters were mainly involved in four categories including binding, catalytic, structural molecule, and transporter activity. Nucleic acid binding, protein binding, and ion binding functions were predominated in the binding category. In the catalytic category, a large number of genes were expressed to produce three kinds of enzymes including oxidoreductases, transferases, and hydrolases. Furthermore, most of the transporters were transmembrane transporters with substrate-specific transporter activities. Cuticular protein genes and ribosomal genes were predominant in the structural molecule category. Significant differences were observed in the clustering of genes among several GO categories. For instance, the cuticular protein genes showed a different distribution in the three clusters, with the majority found in cluster I instead of clusters II and III. Moreover, molecular transducers that function in signal transduction were mainly contained in clusters I and II. By contrast, more of the nucleic acid binding associated genes were expressed like the pattern shown in cluster III. The GO annotation of 6676 genes is provided in Additional file 1.

### Up-regulated and down-regulated genes in epidermal tissues from two stages before ecdysis

To understand better what happens in cuticles when the silkworm initiates a molting cycle, we analyzed the gene expression ratios of IVM to IV4 and W3 to W2, trying to find genes with significant expression changes. The cut-off values were set at a ratio of more than 4 or less than 0.25, which represents up- and down- regulated expression, respectively. As shown in Additional file 2, the expression of ninety-four genes appeared to be more than 4-fold higher in the two molting phases. In this up-regulated gene list, we found sixty-eight cuticular protein genes, four juvenile hormone binding protein genes, two putative genes encoding ecdysteroid regulated proteins, six neuropeptide-like binding protein genes, and two genes related to sclerotization. In contrast, only two genes, *SP1 *and chitinase-related protein 1, were identified with 4-fold less abundant transcripts.

### Two hundred and twenty-seven cuticular protein genes were expressed

**Figure 3:**
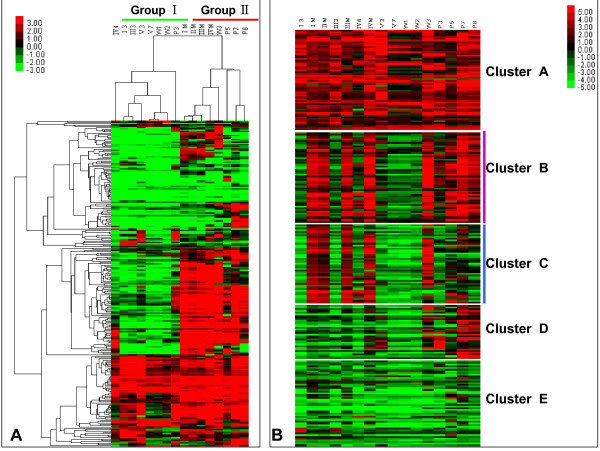
**Heat map of hierarchical clustering of 249 cuticular protein genes in 16 developmental stages**. Clustering was done using Cluster 3 software. The X-fold values of signal intensities divided by 800 were used in clustering. The colors in the map display the relative values of all tiles within the given 16 developmental stages. Green indicates the lowest expression, black indicates the intermediate expression, and red indicates the highest expression. The numerical values give the actual values on a log 2 scale, which were associated with each color. The color scale bar is shown at the top right corner of the figure. **A**: Genes clustered with respect to the 16 developmental stages (clustering type: hierarchical clustering, Distance metric: Pearson correlation). **B**: Genes grouped into five clusters on the basis of the expression similarity (clustering type: K-means clustering, Distance metric: Pearson correlation).

The genome sequencing project for *B. mori *was completed by Chinese and Japanese groups in 2008 [18]. We identified 255 putative cuticular protein genes in an updated silkworm genome database including 151 CPR genes, 4 Tweedle genes, 1 CPF genes, 4 CPFL genes, 51 CPG genes, and 44 CPH genes. Among them, two hundred and twenty-one genes were reported in a previous study [12], the corresponding accession numbers are listed in Additional file 3. Oligonucleotide probes were designed for all except six of the cuticular protein genes, these were *BmorCPR29 *(BR000530; EST: BP117575), *BmorCPR66 *(BR000567; EST: BY914249), *BmorCPG1 *(BR000422), *BmorCPG18 *(BR000439; EST: rswdd0005293.y1.abd), *BmorCPG29 *(BR000450; EST: BP118855), and *BmorCPH29 *(BR000494). In addition, members of three pairs of genes, namely *BmorCPR102 *(BR000603) and *BmorCPR103 *(BR000604), *BmorCPR119 *(BR000620) and *BmorCPR120 *(BR000621), and *BmorCPR124 *(BR000625) and *BmorCPR150 *(GU070697), shared high similarity in the open reading frame (ORF) region, and no unique probes were to distinguish them. Similarly, a single probe was designed to represent three genes, *BmorCPG26 *(BR000447), *BmorCPG27 *(BR000448), and *BmorCPG28 *(BR000449), which shared high identity. In all we obtained 244 oligonucleotide probes representing 249 cuticular protein genes. According to the microarray data, as shown in Figure 3, the expression signals of 227 cuticular protein genes were detected in at least one stage.

### Developmental expression profiles of cuticular protein genes

**Figure 4:**
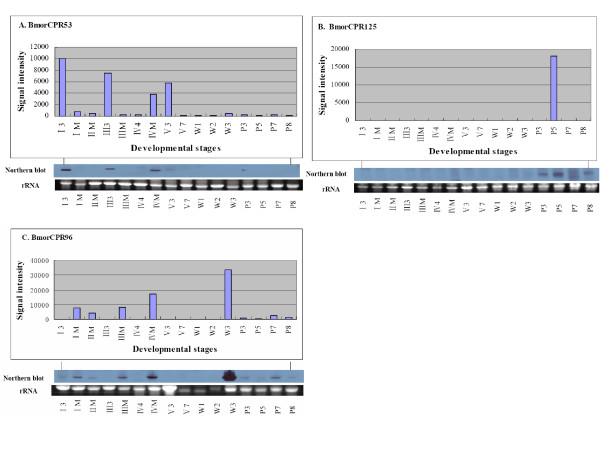
**Expression profiles of three cuticular protein genes**. Top panel, microarray data; middle panel, Northern blot result; lower panel, rRNA loading control. **A**: *BmorCPR53 *(accession number: BR000554), encoding an RR-2 cuticular protein, mainly expressed at molting stages; **B**: *BmorCPR125 *(accession number: BR000626), encoding an RR-2 cuticular protein, specifically expressed in pupal stages; **C**: *BmorCPR96 *(accession number: BR000597), encoding an RR-2 cuticular protein, expressed through larval to pupal stages. 5 μg of total RNA per sample was loaded for northern hybridization, with a wash stringency of 0.1 × SSC.

Hierarchical clustering (Figure 3A) by both samples and genes was performed to examine the expression profiles of cuticular protein genes. The heat map of hierarchical clustering placed 16 developmental stages into two groups. Eight stages, I3, III3, IV4, V3, V7, W1, W2, and P3 were clustered into group I; the remaining stages were in group II. A marked boundary was observed between the two groups. Genes in group I and group II displayed distinctly different expression profiles. Many more cuticular protein genes were expressed in stages which were clustered in group II than in group I. K-means clustering performed to determine the expression patterns of cuticular protein genes yielded five gene clusters with distinct expression patterns, as shown in Figure 3B. Cuticular protein genes contained in cluster A were widely expressed among all 16 developmental stages. Clusters D and E comprised some larval- and pupal- specific expressed cuticular protein genes. Among them, *BmorCPR53 *was only expressed at four stages including I3, III3, IVM, and V3. The northern blot data for this gene (Figure 4A) showed a similar expression pattern at stages I3, III3, and IVM, whereas a weak signal was detected at stage V3. Based on the microarray data, *BmorCPR125 *was identified as a pupal-specific cuticular protein gene expressed at stage P5. The northern blot data (Figure 4B) indicated that except for P5, it was also expressed at a lower level at stages P3, P7, and P8. In clusters B and C, the cuticular protein genes showed a noticeable coordinate expression pattern. However, genes in cluster C had lower expression levels in pupal stages than those in cluster B. Although well organized expression patterns were shown in clusters B and C, exceptions were still found at several stages.

In silkworm, cuticular protein genes are divided into five families based on conserved motifs. To understand better the expression profiles of cuticular protein genes bearing particular motifs, the number of genes in each cluster and the families to which they belong were summarized. As shown in Table 1, each cluster consisted of more than two families. 81.4% (44/54) RR-1 cuticular protein genes were placed into clusters A, B, and E. Among them, 26% (14/54) RR-1 genes found in cluster A were widely expressed in all tested stages. In contrast, a total of 84 (94.5%) RR-2 genes were found in clusters B, C, D, and E. Only 5 (5.5%) RR-2 genes were identified in cluster A. RR-3 genes were found in clusters A and E. Three of four Tweedle cuticular protein genes shared the same expression pattern as cluster B and only one (*BmorCPT1*, BR000650) was found in cluster A with a wide expression pattern. A CPF gene was identified in silkworm but its expression level was too low to be detected. Four CPFL genes were found in clusters A, B, and C. For low complexity cuticular protein genes, 17 CPGs and 19 CPHs were found in cluster A. Altogether, cluster A contained mainly RR-1, CPG, and CPH genes, whereas majority of genes in clusters B and C were RR-1 and RR-2 genes.

**Table 1:** Expression profiles of cuticular protein genes bearing particular motifs

	RR Consensus								
							
	RR-1	RR-2	RR-3	Tweedle	CPF	CPFL	CPG	CPH*	Total
Cluster A	14	5	2	1	0	2	17	19	60
Cluster B	15	21	0	3	0	1	8	6	54
Cluster C	5	32	0	0	0	1	5	4	47
Cluster D	5	13	0	0	0	0	6	8	32
Cluster E	15	18	1	0	1	0	10	6	51
Total	54	89	3	4	1	4	46	43	244

*There is a group of genes (named CPH) where there was only indirect evidence that they coded for possible cuticular proteins.

### Twenty-six RR genes distributed in chromosome 22 were strictly co-expressed in larval and wandering molting stages

**Figure 5:**
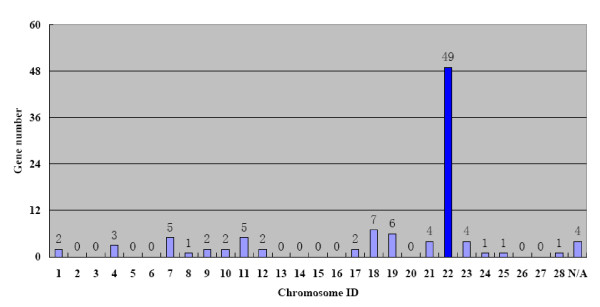
**Chromosomal locations of 101 cuticular protein genes in clusters B and C**. The numbers on the X-axis represent chromosome ID and the numbers on the Y-axis represent the numbers of gene. N/A indicates no available chromosomal location information.

**Figure 6:**
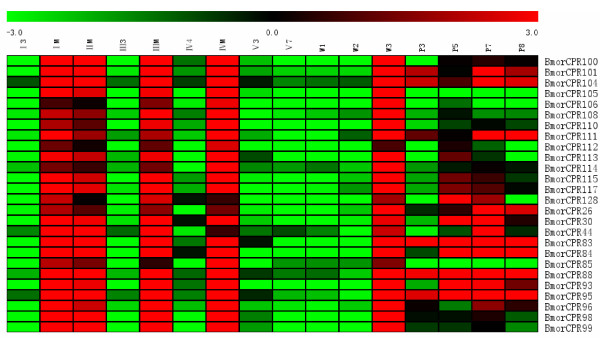
**Heat map showing co-expression during larval and wandering stages of twenty-six RR cuticular protein genes distributed in Chr22**. The X-fold values of signal intensities divided by/800 were used to make the heat map. The colors in map display the relative values of all tiles within the given 16 developmental stages. Green indicates the lowest expression, black indicates the intermediate expression, and red indicates the highest expression. The numerical values give the actual values on a log 2 scale, which were associated with each color. The color scale bar is shown on the top of the figure.

Silkworm cuticular protein genes showed differential patterns of expression, and no obvious correlation was found between expression patterns and the presence of conserved motifs. However, as visualized in the heat map of K-means clustering (Figure 3B), cuticular protein genes in clusters B and C exhibited distinctive expression pattern. Genes in cluster B had abundant transcripts not only in molting stages but also throughout the four pupal stages. The same expression pattern was found for genes in cluster C from stages I3 to W3. But, the number of cuticular protein genes expressed in pupal stages in cluster C appeared to be lower than in cluster B. Considering their similar patterns of expression in larval and wandering stages, we combined the 54 cuticular protein genes in cluster B with the 47 cuticular protein genes in cluster C for the subsequent analysis. First the chromosomal locations of these 101 cuticular protein genes were examined. As shown in Figure 5, 48.5% (49/101) of these genes were distributed in chromosome 22, including 9 RR-1 and 40 RR-2 genes. Stringent QT (quality threshold) clustering algorithm analysis and manual verification were carried out to analyze the expression profiles of these 49 RR cuticular protein genes. Twenty-six of these 49 genes, as shown in Figure 6, were strictly expressed in larval molting stages and in pharate pupae. No expression signals were detected in stages I3, III3, IV4, and V3 to W2. Such result was also supported by the northern hybridization. As shown in Figure 4C, the microarray-based expression profile of *BmorCPR96*, one of these 26 RR genes, was completely consistent with the results of the northern blots.

### Upstream promoter regions of the twenty-six RR genes shared common putative regulatory elements

**Figure 7:**
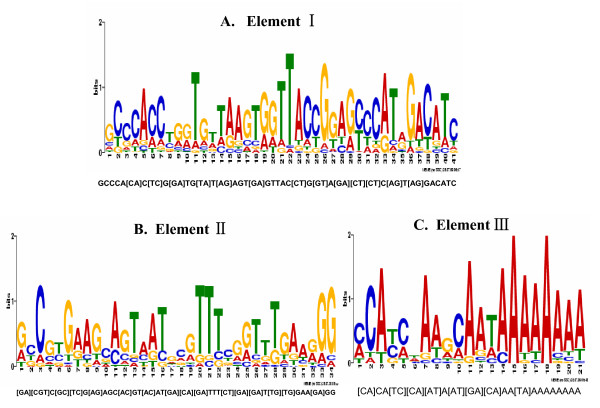
**Elements identified in the promoter regions 2 kb upstream of the transcription start site in 26 cuticular protein genes**. Three elements were shared by all 26 genes. The MEME online sever was used to identify the common elements in the 26 CPR genes. **A**-**C**: Elements I, II, and III containing 41, 34, and 21 nucleotides, respectively.

As noted above, the twenty-six RR cuticular protein genes distributed in chromosome 22 were co-expressed along the larval and wandering stages. To understand the underlying mechanisms of these consistent expression profiles, we analyzed the transcriptional regulatory regions located 2 kb upstream of the putative transcription start sites in the twenty-six genes. The MEME algorithm, which was used to discover similar sequence elements in the promoter regions, identified three putative regulatory elements shared by the 26 RR genes (Figure 7). Elements I, II, and III (Figure 7A-C) contained 41, 34, and 21 nucleotides, respectively. TOMTOM motif comparison tool was employed to compare these three elements with the known motifs. No motifs were found in element II (Figure 7B). Only one NKx2-2 transcription factor binding site was found in the middle of element I (Figure 7A). Nine transcriptional factor binding sites including Hb, Cdx, Nkx6-1, BR-C Z1, BR-C Z2, PLZF, HNF3, FOXP1, and TCF showed similarity with element III (Figure 7C). The alignment match logos for elements I and III are illustrated in Additional file 4. In addition, FIMO (Find Individual Motif Occurrences) was applied to determine whether the elements identified in this study were present in the upstream regions of the cuticular protein genes in *Anopheles gambiae *[19]. Homologues of element II were found upstream of four CPR genes (Additional file 5). Notably, element III was found in the upstream sequences of 59 cuticular protein genes, of which 50 members were CPR genes of *A. gambiae *(Additional file 6).

The TESS (Transcription Element Search System) server was employed to seek known binding sites for transcription factors from the TRANSFAC database in these 2 kb upstream regions [20]. In all the twenty-six upstream sequences, binding sites for at least one of the multiple isoforms of the Broad complex and Ftz-F1 were found. A binding site for the insect ecdysone receptor EcR was also found in the upstream regions of all genes except *BmorCPR83*. In addition, a binding site for the transcription factor E74A was found in the upstream regions of 23 of the 26 cuticular protein genes. Two members of the POU family, Oct-2 and SGF, had binding sites in the upstream regions of 24 and 25 genes, respectively. Nkx2-5, Foxhead, C/EBP, bZIP and bHLH had binding sites in the upstream regions of all 26 RR cuticular protein genes. And binding sites for at least one of the four SGF isoforms were found. The search results also revealed binding sites for B-factor, Dfd, Eve, GATA, Hb, HMG, Pax, Prd, Sox isoforms, TBP, Tll, Twi, Ubx, Zen, Zeste, AP-1, Bcd, and GAGA in the majority of the upstream sequences of these 26 cuticular protein.

## Discussion

Cuticle is generally considered as a protective cover for insects [2-5]. A sclerotized and tanned integument layer consisting of chitin and particular cuticular proteins synthesized and secreted by the epidermal cells play vital roles in insects' lives [1]. In the present study, we monitored gene expression profiles of silkworm epidermal tissues isolated from eleven developmental stages ranging from day 4 of fourth instar larvae to day 8 pupae. In these stages, the expression of 6676 genes was detected. This represents the first global gene expression analysis of Lepidoptera insect epidermal tissues, which provides important functional insight.

Chitin and cuticular proteins are the major components of insect cuticle [21,22]. It is noteworthy to mention that the gene coding for chitin synthase, a critical enzyme for chitin synthesis [23-25], had peak expressions at the same stages in which the majority of cuticular protein genes were also highly expressed, indicating that the processes of cuticle formation were activated concurrently. Our analysis detected the expressions of seven yellow protein genes, several of which are involved in cuticle pigmentation [26-28]. Furthermore, two well-studied melanin syntheses related genes *tyrosine hydroxylase (TH) *and *dopa decarboxylase (DDC) *[29,30], were strongly induced when molting was initiated. These results suggested that many genes involved in cuticle tanning were activated in epidermal tissues before ecdysis. In addition, four juvenile hormone binding protein genes (JHBP) and two ecdysteroid regulated proteins (ERP) were highly expressed before molting. Previous studies showed that JHBPs protect juvenile hormone from degradation [31,32], and the mRNA of *Manduca sexta *ERP20, a homolog of ERP, is abundant in epidermal tissues during molting [33]. Although the functions of these genes have not yet been clarified, it can be assumed that JHBPs and ERPs are involved in the molting process. The expression of six neuropeptide-like protein genes (NPLP) was also stimulated prior to molting. In silkworm, EST evidences showed that neuropeptide-like protein genes were present in epidermis, but their functions were unclear. We speculate that these NPLPs might participate in the regulation of molting. In contrast to numerous up-regulated genes, only two down-regulated genes were detected. SP1 is considered as a storage protein in hemolymph and ovary associated with the development of Lepidoptera insects [34,35]. Another down-regulated gene, chitinase-related protein 1 showed high identity to chitinase. However, without a glutamate residue in the catalytic sites chitinase-related protein 1 did not hydrolyze chitin [36].

The results of the GO analysis presented in Figure 2 showed that genes expressed in epidermal tissues were involved in different pathways, which indicated that cuticle functions not only as a protective cover against external threats but also as a place for active metabolism. In our data, the expressions of silkworm chitinase and genes related to the degradation of proteins were detected. Moreover, numerous transporter genes were expressed in epidermal tissues. One of the conclusions made from these observations was that the synthesis of new cuticle was concomitant with the degradation of the old cuticle. Interestingly, the majority of heat shock protein genes of silkworm were expressed in epidermal tissues throughout the developmental stages, suggesting their role as molecular chaperone was necessary for synthesis and degradation of epidermal proteins [37-39].

Silkworm is a holometabolous insect that develops from larva to pupa, and then pupa to adult. In order to grow and change the appearance, it must molt and cast its old cuticles. So, during molting and metamorphosis dramatic changes occur in cuticles. As the major component of cuticle, cuticular proteins are obvious choices for studying development and metamorphosis. Here, the expressions of cuticular protein genes at 16 stages ranging from day 3 of the first instar larvae to day 8 of pupae were investigated. Of the 244 available probes for cuticular protein gene, 227 genes (93%) had expression signals and were distributed in diverse gene expression patterns. One of goals of this research was to learn which families of cuticular protein genes were expressed in each metamorphic stage. Our data clearly showed no correlation between the expression profiles of cuticular protein genes and the presence of conserved motifs. This result is consistent with what Togawa *et al *found in the expression profile of putative CPR cuticular protein genes of *A. gambiae *by qRT-PCR [14]. On the other hand, our data explicitly revealed massive expression of many cuticular protein genes at molting stages, when the silkworm was building its cuticle. A reasonable explanation for this observation is that these cuticular protein genes were transcribed and immediately translated to proteins participating in the construction of cuticles. Okamoto and colleagues reported that massive numbers of ESTs for *BmorCPR32*, *BmorCPR39 *and *BmorCPG3 *were only isolated during the fourth larval molt, whereas more ESTs of *BmorCPR41 *and *BmorCPR46 *genes were identified in the intermolt stage [40]. In our microarray, *BmorCPR32*, *BmorCPR39*, *BmorCPR41*, and *BmorCPR46 *showed similar patterns to the Okamoto et al.'s [40]. However, *BmorCPG3 *showed a high level at both molting and intermolt stages in our data, which was somewhat different from the patterns of Okamoto *et al*. This can be explained by the differences in sensitivity between EST sequencing and microarrays. Our data also revealed that although the cuticles of silkworm larva, pupa, and adult were distinct, a number of cuticular protein genes were commonly expressed at all stages, indicating that the properties of cuticle depend on the amount of cuticular proteins, and their spatial distribution, the degree of sclerotization and tanning [1], rather than simply the types of cuticular proteins present.

The silkworm used more than 1.5% of its total estimated genes to encode the cuticular proteins, and at each molting stage massive cuticular protein genes were activated. Another goal of this research was to learn how the silkworm regulates the transcription of cuticular protein genes. Coordinate regulation using the same conserved motifs or localizing genes on the same chromosomes might be the simplest and most efficient mechanism. Our data revealed no evidence for coordinate regulation in the majority of cuticular protein genes. However, twenty-six RR cuticular protein genes co-expressed in larval stages were found to be distributed in chromosome 22. Togawa and colleagues also found that a portion of *A. gambiae *cuticular protein genes distributed in a narrow chromosome region showed highly similar expression patterns [14]. Three similar elements were further identified in the 2 kb upstream promoter regions of the 26 silkworm cuticular genes on chromosome 22. Notably, the similar sequences of element II and III were found in the upstream regions of *A. gambiae *CPR genes, and a binding site of transcriptional factor NKx2-2 was identified in the middle of element. Nkx2-2 acts cooperatively with Pax6, whose function was conserved from invertebrate to vertebrate for dorsal and ventral patterning [41,42]. A recent study showed that Broad-Complex and βFTZ-F1 positively regulated the transcription of wing cuticular protein gene in silkworm [43]. Interestingly, element III found in the present study was much longer than the binding sequence of any Broad Complex isoform. The three elements might account for the coordinate regulation of the 26 cuticular protein genes. Future analysis of these elements will increase the understanding of transcriptional regulation of cuticular protein genes.

Besides, many binding sites for known transcription factors were identified in the upstream regions of these cuticular protein genes. Binding sites for EcR and E74A were discovered in the upstream regions of 25, and 23 of the 26 cuticular protein genes, respectively. EcR formed heterodimer with USP (ultraspiracle protein) to function as the receptor of ecdysone and E74A was known as the early genes induced by ecdysone and functioned as one of ecdysone signal transducers [44-46]. Binding sites for bZIP, bHLH, and C/EBP were discovered in upstream sequences of all 26 cuticular protein genes. Previous study showed that transcription factors with bZIP and bHLH domain played roles in transcriptional regulation of neuropeptides and peptide hormone [47], which were engaged in the regulation of insect molting. C/EBP was the factor bound to the promoters of silkworm chorion genes and regulated their precise spatial and temporal expression [48,49]. In silkworm, POU factors played a critical role in transcriptional regulation of neuropeptides and silk genes [50-52].

Generally, the expression of cuticular protein genes is regulated by two hormones, ecdysone and juvenile hormone. Ecdysone induces the transcription of the primary-response genes [46,53], including Broad Complex genes, E74 isoforms, βFTZ-F1, and orphan nuclear receptors. Primary-response genes activate and regulate the transcription of the secondary-response target genes. Although a number of juvenile hormone binding proteins have been identified [31,32], little was known about the juvenile hormone receptor. Cuticular protein genes are usually considered to be located downstream of the hormones action hierarchy [44]. The transcription of cuticular protein genes could be activated by various signaling pathways, which provides a possible explanation for the diverse expression patterns demonstrated in this study.

## Conclusions

This study describes the expression profile of genes in silkworm epidermal tissues for the first time. Microarray data showed activation of a total of 6676 genes involved in various functions and physiological pathways. More than 93% cuticular protein genes were expressed in selected developmental stages, displaying diverse expression patterns. The majority of cuticle proteins showed no evidence of coordinate regulation as a function of common cuticle protein motifs. However, 26 RR genes distributed in chromosome 22 were co-expressed at larval and wandering stages, and three putative elements were identified in the 2 kb upstream region of these 26 RR genes. Extensive expression data and the analysis of transcriptional factor binding sites provided novel insights into the functional coordination of these genes.

## Methods

### Microarray design and construction

Based on a previously designed silkworm oligonucleotide microarray [15], we added 147 novel oligonucleotide probes for the cuticular protein genes that were not present in the original array. All the probes were designed by CapitalBio Corporation (Beijing, China) and were synthesized by MWG Biotech (Ebersberg, Germany). The microarray slide contained 48 blocks, each with 22 rows and 23 columns. Five housekeeping genes and eight yeast intergenic sequences were dotted in one block as positive and external controls, respectively. Dual channel microarray hybridization was performed with a Cy3-labeled control sample and Cy5-labeled test sample. Total RNAs extracted from the whole body of silkworm at day 3 of the fifth instar larvae served as a normalization control for data analysis.

### Silkworm strain and reagents

Silkworm larvae (p50 strain) maintained at the Institute of Sericulture and System Biology (Southwest University, China) were reared on mulberry leaves at 25°C~26°C. Silkworms grow through five instars until cocoon spinning which begins at the end of the fifth instar larva day 7. After spinning for three days, silkworms develop into the pupal stage, which takes about 10 days, followed by emergence from the cocoon, mating and egg lay. We selected sixteen time points around the molting phases, ranged from the first instar larva day 3 to pupa day 8. Considering the small body size, we used whole larval bodies from the first to the third instar larvae to isolate total RNA. From the fourth instar larva day 4 to pupa day 8, we collected epidermal tissues to isolate total RNA. TRIzol reagent was obtained from Invitrogen (Carlsbad, CA, USA). Reverse transcriptase was made in Promega (Madison, WI, USA). ECL direct nucleic acid labeling and detection system was from GE Healthcare (Buckinghamshire, UK).

### RNA isolation, amplification, labeling and array hybridizations

Total RNAs were isolated using TRIzol reagent and further purified using a NucleoSpin RNA clean-up kit (Macherey-Nagel, Germany). The amplification and labeling of mRNA were performed as described in previous studies [15,54]. Five micrograms of total RNA were primed with 1 μl of 100 μM primer containing T7 RNA polymerase promoter sequence at 70°C for 10 min, then reversed transcribed at 42°C for 2 h in the presence of 200 U CbcScript (CapitalBio Corp, China). The second strand of cDNA was synthesized at 16°C for 2 h in the presence of RNaseH and DNA polymerase. cRNA was synthesized by T7 Enzyme Mix (CapitalBio Corp, China) using the cDNA template. 2 μl of cRNA were primed with 1 μl random primer at 65°C for 10 min, then reverse transcribed at 25°C for 10 min and 37°C for 1.5 h in the presence of CbcScript II (CapitalBio Corp, China). The Cy3- and Cy5-dCTP double-stranded cDNA was labeled using a CapitalBio cRNA Amplification and Labeling Kit (CapitalBio, Beijing, China). Cy5-dCTP or Cy3-dCTP were added at a final concentration of 120 μM of each dATP, dGTP, and dTTP and 60 μM dCTP and 40 μM Cy5-dCTP for test samples. For reference samples, Cy3-dCTP was used. The Cy3- and Cy5-dCTP double-stranded cDNA was dissolved in 80 μl hybridization solution containing 3 × SSC, 0.2%SDS, 5 × Denhart's, and 25% formamide. The slides were covered with a LifterSlip™ coverslip (Erie Company, Portsmouth, NH, USA) and hybridized in a closed chamber at 42°C over-night. After hybridization, slides were washed three times in 0.2% SDS, 2 × SSC at 42°C for 5 minutes and three times in 0.2 × SSC at room temperature for 5 minutes before signal scanning.

### Microarray data processing and analysis

The slides were scanned with a confocal LuxScan scanner (CapitalBio Corp.) and the raw data were extracted using LuxScan™ 3.0 software (CapitalBio Corp.). For dual-channels microarray data, the scanning setting for Cy3 and Cy5 channels were balanced by visual inspection of the external control spots. The LOWESS (Locally Weighted Scatterplot Smoothing) method was used to normalize the dual channel data using all the signals from the Cy3-labeled sample. The ratios of signal intensity of test and control samples were used to perform clustering analysis. The one with a fluorescence intensity higher than 800 after subtracting the background was considered as an expressed gene since the signal greater than that detection level was more reliable. The expression of a cuticular protein gene was defined by the ratio of the original signal intensity divided by 800. The X-fold values were used in the subsequent clustering analysis to display the expression of cuticular protein genes at different developmental stages. HCL (Hierarchical Clustering) analysis was carried out using both Cluster 3.0 software and Mev software (version 4.2.01) [55,56]. In addition, Cluster 3.0 software was used for K-mean clustering analysis. Mev software was also used for QT (quality threshold) clustering. The parameter setting for clustering analysis was based on the distance metric of the Pearson correlation and the average linkage method. TreeView software was used to display heat map of clustering results. Gene ontology analysis was performed at the BGI WEGO website [57].

### Computational identification of putative regulatory elements

The MEME algorithm (Multiple Expectation maximization for Motif Elicitation) was used to identify common elements present in the 2 kb promoter regions upstream of the transcription start sites of cuticular protein genes [58]. TOMTOM motif comparison tool was used to compare the elements identified in this study to known motifs [59]. In TOMTOM analysis, the TRANSFAC database was selected and the Pearson correlation coefficient was employed to survey the Motif Column Comparison Function. FIMO (Find Individual Motif Occurrences) was applied to search for whether the identified regulatory elements existed upstream of other genes [19]. In FIMO analysis, the *Anopheles_gambiae*_EnsEMBL_upstream database was selected as the reference and the *p*-value output threshold was set at l × e-5. TESS (Transcription Element Search System) was applied to search the binding sites for known insect transcription factors from the TRANSFAC database [20].

### Northern hybridization

Northern hybridization was performed to confirm the microarray data. The sequences of cuticular protein genes used to design the hybridization probes were obtained from the Silkworm Genome Database [17]. DEPC water was employed to prepare the related solutions and to clean the associated equipments. Five micrograms total RNA per sample was loaded to perform denaturing formaldehyde gel electrophoresis. The transfer of RNA from gel to Hybond+ (GE) membrane was completed in 2 hr by using Transfer Equipment (Amersham Biosciences). All reagents used in prehybridization, probes labeling, hybridization and signal detection were provided by the Amersham ECL Direct Nucleic Acid Labeling and Detection Systems (GE Healthcare, Cat No: PRN 3001), which is based on enhanced chemiluminescence. The optimized temperature of hybridization mixture 42°C was adopted to protect the activity of the horseradish peroxidase. The cDNA probes, which were labeled with the enzyme horseradish peroxidase, were completely denatured to single-strand form to hybridize the target RNA on Hybond+ membrane. Membranes were washed to a stringency of 0.1 × SSC, and labeling and detection were carried out according to the manufacturer's instructions.

## Abbreviations

I3: day 3 of the first instar larva; IM: larval molting stage from 1^st ^to 2^nd ^instar; IIM: larval molting stage from 2^nd ^to 3^rd ^instar; III3: day 3 of the third instar larva; IIIM: larval molting stage from 3^rd ^to 4^th ^instar; IV4: day 4 of the fourth instar larva; IVM: larval molting stage from 4^th ^to 5^th ^instar; V3: day 3 of the fifth instar larva; V7: day 7 of the fifth instar larva; W1: day 1 of the wandering phase; W2: day 2 of the wandering phase; W3: day 3 of the wandering phase; P3: day 3 of the pupa; P5: day 5 of the pupa; P7: day 7 of the pupa; P8: day 8 of the pupa; CPF: cuticular protein with 44-amino acid motif; CPFL: CPF-like protein; CPG: glycine-rich cuticular protein; CPH: hypothetical cuticular protein; CPR: cuticular protein with the R&R Consensus; CPT: cuticular protein with a Tweedle motif; GO: gene ontology; QT clustering: quality threshold clustering; MEME: multiple expectation maximization for motif elicitation (motif discovery tool); TOMTOM: motif comparison tool for searching a database of motifs with a given query motif; FIMO: find individual motif occurrences; TESS: transcription element search system.

## Authors' contributions

NH conceived of the study and developed the study design. JL and LZ carried out the analysis and drafted the manuscript. ZX participated in the study design and drafting of the manuscript. NH and LZ contributed to the critical revision of the manuscript. All authors read and approved the final manuscript.

## Supplementary Material

Additional file 1 **The GO (Gene Ontology) annotation of 6676 probes**. This file contains the GO number of 6676 probes expressed in our microarray. It has multiple columns--the first of which is the probe ID and the rest with GO numbers. Some probes representing new genes have no GO number. Click here for file

Additional file 2 **The list of ninety-four up-regulated and two down-regulated genes from two stages before ecdysis**. This file contains the ninety-four up-regulated and two down-regulated genes from two stages before ecdysis. It has a table which contains five columns. The first column is the function terms which these genes belong to; the second to the fifth with names of these genes, symbol or homolog, accession number and E-value, respectively. Click here for file

Additional file 3 **The cuticular protein genes in silkworm**. It is a table that contains all the silkworm cuticular protein genes. The gene families belonging, gene symbol or names, and accession numbers are also given. Click here for file

Additional file 4 **The alignment match logos of elements I and III with known transcription factors**. This file contains two figures illustrating the alignment match logos of elements Iand III with known transcription factors. In each figure, the name of transcription factors and relative parameters are given. Click here for file

Additional file 5 **List of genes of *Anopheles gambiae *sharing homologous sequences with elements II**. This file contains *Anopheles gambiae *genes which share homologous sequences with elements II identified in this study. The gene ID and some parameters also contained. Click here for file

Additional file 6 **List of genes of *Anopheles gambiae *sharing homologous sequences with elements III**. This file contains the genes of *Anopheles gambiae *sharing homologous sequences with elements III identified in this study. The gene ID and some parameters also contained. Click here for file

## References

[B1] Andersen SO Gilbert LI, Iatrou K, Gill S Cuticular Sclerotization and Tanning Comprehensive Molecular Insect Science 2005 4 Oxford: Elsevier Pergamon Press 145 170 full_text

[B2] Chapman RF The Insects: Structure and Function 1969 London: University Press Ltd

[B3] Weber H Weidner H Grundniss der Insectenkunde 1974 Stuttgart: Gustav Fischer Verlag

[B4] Fretter V Graham A A Functional Anatomy of Invertebrates 1976 Academic Press, London, New-York, San Francisco

[B5] Meglitsch PA Schram FR Invertebrate Zoology 1991 New York: Oxford University Press

[B6] Willis JH Iconomidou VA Smith RF Hamodrakas SJ Gilbert LI, Iatrou K, Gill S Cuticular proteins Comprehensive Molecular Insect Science 2005 4 Oxford: Elsevier Pergamon Press 79 109 full_text

[B7] Rebers J Riddiford LM Structure and expression of a *Manduca sexta *larval cuticle gene homologous to *Drosophila *cuticle genes J Mol Biol 1988 203 411 423 10.1016/0022-2836(88)90009-5 2462055

[B8] Togawa T Dunn WA Emmons AC Willis JH CPF and CPFL, two related gene families encoding cuticular proteins of *Anopheles gambiae *and other insects Insect Biochem Molec BioL 2007 37 675 688 10.1016/j.ibmb.2007.03.011 17550824

[B9] Guan X Middlebrooks BW Alexander S Wasserman SA Mutation of TweedleD, a member of an unconventional cuticle protein family, alters body shape in *Drosophila* Proc Natl Acad Sci USA 2006 103 16794 16799 10.1073/pnas.0607616103 17075064 PMC1636534

[B10] Cornman RS Togawa T Dunn WA He NJ Emmons AC Willis JH Annotation and analysis of a large cuticular protein family with the R&R Consensus in *Anopheles gambiae* BMC Genomics 2008 9 22 10.1186/1471-2164-9-22 18205929 PMC2259329

[B11] Karouzou MV Spyropoulos Y Iconomidou VA Cornman RS Hamodrakas SJ Willis JH *Drosophila *cuticular proteins with the R&R Consensus: annotation and classification with a new tool for discriminating RR-1 and RR-2 sequences Insect Biochem Mol Biol 2007 37 754 760 10.1016/j.ibmb.2007.03.007 17628275

[B12] Futahashi R Okamoto S Kawasaki H Zhong YS Iwanaga M Mita K Fujiwara H Genome-wide identification of cuticular protein genes in the silkworm, *Bombyx mori* Insect Biochem Mol Biol 2008 38 1138 1146 10.1016/j.ibmb.2008.05.007 19280704

[B13] The Honeybee Genome Sequencing Consortium Insights into social insects from the genome of the honeybee *Apis mellifera* Nature 2006 443 931 949 10.1038/nature05260 17073008 PMC2048586

[B14] Togawa T Dunn WA Emmons AC Nagao J Willis JH Developmental expression patterns of cuticular protein genes with the R&R Consensus from *Anopheles gambiae* Insect Biochem Mol Biol 2008 38 508 519 10.1016/j.ibmb.2007.12.008 18405829 PMC2416445

[B15] Xia QY Cheng DJ Duan J Wang GH Cheng TC Zha XF Liu C Zhao P Dai FY Zhang Z He NJ Zhang L Xiang ZH Microarray-based gene expression profiles in multiple tissues of the domesticated silkworm, *Bombyx mori* Genome Biology 2007 8 R162 10.1186/gb-2007-8-8-r162 17683582 PMC2374993

[B16] Xia QY Zhou ZY Lu C Cheng DJ Dai FY Li B Zhao P Zha XF Cheng TC Chai CL Pan GQ Xu JS Liu C Lin Y Qian JF Hou Y Wu ZL Li GR Pan MH Li CF Shen YH Lan XQ Yuan LW Li T Xu HF Yang GW Wan YJ Zhu Y Yu MD Shen WD A draft sequence for the genome of the domesticated silkworm (*Bombyx mori*) Science 2004 306 1937 1940 10.1126/science.1102210 15591204

[B17] Silkworm Genome Databese http://silkworm.swu.edu.cn/silkdb/

[B18] The International Silkworm Genome Consortium The genome of a lepidopteran model insect, the silkworm *Bombyx mori* Insect Biochem Mol Biol 2008 38 1036 1045 10.1016/j.ibmb.2008.11.004 19121390

[B19] FIMO sever http://meme.nbcr.net/meme/cgi-bin/fimo.cgi

[B20] TESS sever http://www.cbil.upenn.edu./cgi-bin/tess/tess

[B21] Willis JH Cuticular proteins: The neglected component Arch Insect Biochem Physiol 1987 6 203 215 10.1002/arch.940060402

[B22] Andersen SO Højrup P Roepstorff P Insect cuticular proteins Insect Biochem Mol Biol 1995 25 153 176 10.1016/0965-1748(94)00052-J 7711748

[B23] Kramer KJ Koga D Insect chitin: physical state, synthesis, degradation and metabolic regulation Insect Biochem 1986 16 851 877 10.1016/0020-1790(86)90059-4

[B24] Cohen E Chitin synthesis and inhibition: a revisit Pest Manag Sci 2001 57 946 950 10.1002/ps.363 11695188

[B25] Merzendorfer H Zimoch L Chitin metabolism in insects: structure, function and regulation of chitin synthases and chitinases J Exp Biol 2003 206 4393 4412 10.1242/jeb.00709 14610026

[B26] Wittkopp PJ True JR Carroll SB Reciprocal functions of the *Drosophila *Yellow and Ebony proteins in the development and evolution of pigment patterns Development 2002 129 1849 1858 11934851 10.1242/dev.129.8.1849

[B27] Xia AH Zhou QX Yu LL Li WG Yi YZ Zhang YZ Zhang ZF Identification and analysis of YELLOW protein family genes in the silkworm, *Bombyx mori* BMC Genomics 2006 7 195 10.1186/1471-2164-7-195 16884544 PMC1553450

[B28] Futahashi R Sato J Meng Y Okamoto S Daimon T Yamamoto K Suetsugu Y Narukawa J Takahashi H Banno Y Katsuma S Shimada T Mita K Fujiwara H *yellow *and *ebony *are the responsible genes for the larval color mutants of the silkworm *Bombyx mori* Genetics 2008 180 1995 2005 10.1534/genetics.108.096388 18854583 PMC2600937

[B29] Futahashi R Fujiwara H Melanin-synthesis enzymes coregulate stage-specific larval cuticular markings in the swallowtail butterfly, *Papilio xuthus* Dev Genes Evol 2005 215 519 529 10.1007/s00427-005-0014-y 16133568

[B30] Davis MM O'Keefe SL Primrose DA Hodgetts RB A neuropeptide hormone cascade controls the precise onset of post-eclosion cuticular tanning in *Drosophila melanogaster* Development 2007 134 4395 4404 10.1242/dev.009902 18003740

[B31] Kort CAD Granger NA Regulation of JH titers: The relevance of degradative enzymes and binding proteins Arch Insect Biochem Physiol 1996 33 1 26 10.1002/(SICI)1520-6327(1996)33:1<1::AID-ARCH1>3.0.CO;2-2

[B32] Goodman WG Granger NA Gilbert LI, Iatrou K, Gill S The juvenile hormones Comprehensive Molecular Insect Science 2005 3 Oxford: Elsevier Pergamon Press 319 408 full_text

[B33] Mészáros M Morton DB Isolation and partial characterization of a gene from trachea of *Manduca sexta *that requires and is negatively regulated by Ecdysteroids Dev Biol 1994 162 618 630 10.1006/dbio.1994.1115 8150220

[B34] Tungjitwitayakul J Singtripop T Nettagul A Oda Y Tatun N Sekimoto T Sakurai S Identification, characterization, and developmental regulation of two storage proteins in the bamboo borer *Omphisa fuscidentalis* Journal Insect Physiol 2008 154 62 76 10.1016/j.jinsphys.2007.08.003 17869264

[B35] Chandrasekar R Jae SS Krishnan M Expression and Localization of Storage Protein 1 (SP1) in Differentiated Fat Body Tissues of Red Hairy Caterpillar, *Amsacta albistriga *Walker Arch Insect Biochem Physiol 2008 69 70 84 10.1002/arch.20266 18780375

[B36] Takahashi M Kiuchi M Kamimura M A new chitinase-related gene, BmChiR1, is induced in the *Bombyx mori *anterior silk gland at molt and metamorphosis by ecdysteroid Insect Biochem Mol Biol 2002 32 147 151 10.1016/S0965-1748(01)00102-3 11755056

[B37] Lindquist S Craig EA The heat-shock proteins Annu Rev Genet 1988 22 631 677 10.1146/annurev.ge.22.120188.003215 2853609

[B38] Jakob U Gaestel M Engel K Buchner J Small heat shock proteins are molecular chaperones J Biol Chem 1993 268 1517 1520 8093612

[B39] Van Montfort RLM Basha E Friedrich KL Slingsby C Vierling E Crystal structure and assembly of a eukaryotic small heat shock protein Nat Struct Mol Biol 2001 8 1025 1030 10.1038/nsb722 11702068

[B40] Okamoto S Futahashi R Kojima T Mita K Fujiwara H Catalogue of epidermal genes: Genes expressed in the epidermis during larval molt of the silkworm *Bombyx mori* BMC Genomics 2008 9 396 10.1186/1471-2164-9-396 18721459 PMC2542385

[B41] Briscoe J Sussel L Serup P Hartigan-O'Connor D Jessell TM Rubenstein JL Ericson J Homeobox gene Nkx2.2 and specification of neuronal identity by graded Sonic hedgehog signalling Nature 1999 398 622 627 10.1038/19315 10217145

[B42] Ericson J Rashbass P Schedl A Brenner-Morton S Kawakami A Van Heyningen V Jessell TM Briscoe J Pax6 controls progenitor cell identity and neuronal fate in response to graded Shh signaling Cell 1997 90 169 180 10.1016/S0092-8674(00)80323-2 9230312

[B43] Wang HB Nita M Iwanaga M Kawasaki H βFTZ-F1 and Broad-Complex positively regulate the transcription of the wing cuticle protein gene, BMWCP5, in wing discs of *Bombyx mori* Insect Biochem Mol Biol 2009 39 624 633 10.1016/j.ibmb.2009.06.007 19580866

[B44] Fletcher JC Thummel CS The *Drosophila *E74 gene is required for the proper stage- and tissue-specific transcription of ecdysone regulated genes at the onset of metamorphosis Development 1995 121 1411 1421 7789271 10.1242/dev.121.5.1411

[B45] Fletcher JC D'Avino PP Thummel CS A steroid-triggered switch in E74 transcription factor isoforms regulates the timing of secondary-response gene expression Proc Natl Acad Sci USA 1997 94 4582 4586 10.1073/pnas.94.9.4582 9114033 PMC20766

[B46] Buszczak M Segraves WA Insect metamorphosis: out with the old, in with the new Curr Biol 2000 10 R830 R833 10.1016/S0960-9822(00)00792-2 11102824

[B47] Gauthier SA Hewes RS Transcriptional regulation of neuropeptide and peptide hormone expression by the *Drosophila dimmed *and *cryptocephal *genes J Exp Biol 2006 209 1803 1815 10.1242/jeb.02202 16651547

[B48] Kafatos FC Tzertzinis G Spoerel NA Nguyen HT Goldsmith MR and Wilkins AS Chorion genes: an overview of their structure, function and transcriptional regulation Molecular Model System in the Lepidoptera 1995 London: Cambridge University Press 181 216 full_text

[B49] Sourmeli S Papantonis A Lecanidou R A novel role for the *Bombyx *Slbo homologue, BmC/EBP, in insect choriogenesis Biochem Biophys Res Commun 2005 337 713 719 10.1016/j.bbrc.2005.09.103 16202393

[B50] Zhang TY Kang L Zhang ZF Xu WH Identification of a POU factor involved in regulating the neuron-specific expression of the gene encoding diapause hormone and pheromone biosynthesis-activating neuropeptide in *Bombyx mori* Biochem J 2004 380 255 263 10.1042/BJ20031482 14766018 PMC1224146

[B51] Fukuta M Matsuno K Hui CC Nagata T Takiya S Xu PX Ueno K Suzuki Y Molecular cloning of a POU domain-containing factor involved in the regulation of the *Bombyx *sericin-1 gene J Biol Chem 1993 268 19471 19475 7690034

[B52] Matsunami K Kokubo H Ohno K Suzuki Y Expression pattern analysis of SGF-3/POU-M1 in relation to sericin-1 gene expression in the silk gland Dev Growth Differ 1998 40 591 597 10.1046/j.1440-169X.1998.00398.x 9865969

[B53] Thummel CS Flies on steroids---*Drosophila *metamorphosis and the mechanisms of steroid hormone action Trends Genet 1996 12 306 310 10.1016/0168-9525(96)10032-9 8783940

[B54] Patterson TA Lobenhofer EK Fulmer-Smentek SB Collins PJ Chu TM Bao WJ Fang H Kawasaki ES Hager J Tikhonova IR Walker SJ Zhang L Hurban P Longueville F Fuscoe JC Tong WD Shi L Wolfinger RD Performance comparison of one-color and two-color platforms within the MicroArray Quality Control (MAQC) project Nat Biotechnol 2006 24 1140 1150 10.1038/nbt1242 16964228

[B55] Eisen MB Spellman PT Brown PO Botstein D Cluster analysis and display of genome-wide expression patterns Proc Natl Acad Sci USA 1998 95 14863 14868 10.1073/pnas.95.25.14863 9843981 PMC24541

[B56] Saeed AI Sharov V White J Li J Liang W Bhagabati N Braisted J Klapa M Currier T Thiagarajan M Sturn A Snuffin M Rezantsev A Popov D Ryltsov A Kostukovich E Borisovsky I Liu Z Vinsavich A Trush V Quackenbush J TM4: a free, open-source system for microarray data management and analysis Biotechniques 2003 34 374 378 12613259 10.2144/03342mt01

[B57] BGI WEGO http://wego.genomics.org.cn/cgi-bin/wego/index.pl GO Archive: 2008-10-01

[B58] MEME sever http://meme.nbcr.net/meme/cgi-bin/meme.cgi

[B59] TOMTOM sever http://meme.nbcr.net/meme/cgi-bin/tomtom.cgi

