# Supplementary material for: Expression profile of cuticular genes of silkworm, Bombyx mori
Source: BMC Genomics. 2010 Mar 15;11:173. doi: 10.1186/1471-2164-11-173 (PMC2848646; doi:10.1186/1471-2164-11-173)
Supplement: Additional file 2 — The list of ninety-four up-regulated and two down-regulated genes from two stages before ecdysis. This file contains the ninety-four up-regulated and two down-regulated genes from two stages before ecdysis. It has a table which contains five columns. The first column is the function terms which these genes belong to; the second to the fifth with names of these genes, symbol or homolog, accession number and E-value, respectively. [file 1471-2164-11-173-S2.DOC]

Additional file 2: List of ninety-four up-regulated and two down-regulated genes

| **Up-regulated genes** |  |  |  |  |
| --- | --- | --- | --- | --- |
| Function | Gene name | Symbol/homolog | Accession no | E-value |
| Cuticular protein | Bm_nscaf2964_132 | BmorCPFL1 | BR000418 | 0 |
|  | BGIBMGA011559 | BmorCPFL2 | BR000419 | 0 |
|  | BGIBMGA011560 | BmorCPFL3 | BR000420 | 0 |
|  | BGIBMGA011455 | BmorCPFL4 | BR000421 | 0 |
|  | BGIBMGA002385 | BmorCPG12 | BR000433 | 0 |
|  | BGIBMGA002384 | BmorCPG13 | BR000434 | 0 |
|  | BGIBMGA011561 | BmorCPG23 | BR000444 | 0 |
|  | BGIBMGA009659 | BmorCPH2 | BR000452 | 0 |
|  | BGIBMGA009776 | BmorCPH4 | BR000454 | 0 |
|  | BGIBMGA011731 | BmorCPH8 | BR000463 | 0 |
|  | BGIBMGA011730 | BmorCPH10 | BR000465 | 0 |
|  | BGIBMGA011729 | BmorCPH11 | BR000466 | 0 |
|  | BGIBMGA011725 | BmorCPH16 | BR000471 | 0 |
|  | BGIBMGA011723 | BmorCPH18 | BR000473 | 0 |
|  | BGIBMGA011721 | BmorCPH21 | BR000476 | 0 |
|  | BGIBMGA011719 | BmorCPH23 | BR000478 | 0 |
|  | BGIBMGA010500 | BmorCPH28 | BR000493 | 0 |
|  | BGIBMGA012861 | BmorCPH30 | BR000495 | 0 |
|  | Bm_nscaf3058_162 | BmorCPH31 | BR000496 | 0 |
|  | BGIBMGA005277 | BmorCPR2 | BR000503 | 0 |
|  | BGIBMGA002548 | BmorCPR4 | BR000505 | 0 |
|  | BGIBMGA002549 | BmorCPR5 | BR000506 | 0 |
|  | BGIBMGA007015 | BmorCPR8 | BR000509 | 0 |
|  | BGIBMGA008333 | BmorCPR10 | BR000511 | 0 |
|  | BGIBMGA012605 | BmorCPR15 | BR000516 | 0 |
|  | BGIBMGA000340 | BmorCPR30 | BR000531 | 0 |
|  | BGIBMGA000338 | BmorCPR32 | BR000533 | 0 |
|  | BGIBMGA000332 | BmorCPR39 | BR000540 | 0 |
|  | BGIBMGA000327 | BmorCPR43 | BR000544 | 0 |
|  | BGIBMGA003065 | BmorCPR59 | BR000560 | 0 |
|  | BGIBMGA003062 | BmorCPR63 | BR000564 | 0 |
|  | BGIBMGA010145 | BmorCPR67 | BR000568 | 0 |
|  | BGIBMGA010231 | BmorCPR68 | BR000569 | 0 |
|  | BGIBMGA010232 | BmorCPR69 | BR000570 | 0 |
|  | BGIBMGA010143 | BmorCPR70 | BR000571 | 0 |
|  | BGIBMGA010142 | BmorCPR71 | BR000572 | 0 |
|  | BGIBMGA002362 | BmorCPR75 | BR000576 | 0 |
|  | BGIBMGA001486 | BmorCPR76 | BR000577 | 0 |
|  | BGIBMGA001487 | BmorCPR77 | BR000578 | 0 |
|  | BGIBMGA000281 | BmorCPR83 | BR000584 | 0 |
|  | BGIBMGA000280 | BmorCPR84 | BR000585 | 0 |
|  | BGIBMGA000278 | BmorCPR86 | BR000587 | 0 |
|  | BGIBMGA000277 | BmorCPR87 | BR000588 | 0 |
|  | BGIBMGA000276 | BmorCPR88 | BR000589 | 0 |
|  | BGIBMGA000273 | BmorCPR91 | BR000592 | 0 |
|  | BGIBMGA000271 | BmorCPR93 | BR000594 | 0 |
|  | BGIBMGA000269 | BmorCPR95 | BR000596 | 0 |
|  | BGIBMGA000268 | BmorCPR96 | BR000597 | 0 |
|  | BGIBMGA000267 | BmorCPR97 | BR000598 | 0 |
|  | BGIBMGA000266 | BmorCPR98 | BR000599 | 0 |
|  | BGIBMGA000265 | BmorCPR99 | BR000600 | 0 |
|  | BGIBMGA000264 | BmorCPR100 | BR000601 | 0 |
|  | BGIBMGA000428 | BmorCPR101 | BR000602 | 0 |
|  | BGIBMGA000429 | BmorCPR103 | BR000604 | 0 |
|  | BGIBMGA000430 | BmorCPR104 | BR000605 | 0 |
|  | BGIBMGA000263 | BmorCPR105 | BR000606 | 0 |
|  | BGIBMGA000262 | BmorCPR106 | BR000607 | 0 |
|  | BGIBMGA000431 | BmorCPR108 | BR000609 | 0 |
|  | BGIBMGA000432 | BmorCPR110 | BR000611 | 0 |
|  | BGIBMGA000433 | BmorCPR111 | BR000612 | 0 |
|  | BGIBMGA000258 | BmorCPR113 | BR000614 | 0 |
|  | BGIBMGA000257 | BmorCPR114 | BR000615 | 0 |
|  | BGIBMGA000256 | BmorCPR115 | BR000616 | 0 |
|  | BGIBMGA000255 | BmorCPR117 | BR000618 | 0 |
|  | BGIBMGA000250 | BmorCPR126 | BR000627 | 0 |
|  | BGIBMGA014295 | BmorCPR138 | BR000639 | 0 |
|  | *BGIBMGA000252/ Bm_nscaf1681_194 | *BmorCPR124 **/** BmorCPR150 | *BR000625/ GU070697 | 0 |
|  | BGIBMGA006826 | BmorCPT4 | BR000653 | 0 |
| Putative JHBP | BGIBMGA011556 | Takeout-like protein 3 | ACF39403 | 3.00E-46 |
|  | BGIBMGA001308 | JHBP | BAH97092 | 2.00E-38 |
|  | BGIBMGA011457 | JHBP | BAH97100 | 1.00E-32 |
|  | BGIBMGA011458 | JHBP | BAH97095 | 3.00E-146 |
| Putative ERP | BGIBMGA010977 | ESR20 | AAA29312 | 2.00E-08 |
|  | BGIBMGA010985 | ESR20 | AAA29312 | 9.00E-44 |
| Melanization related | BGIBMGA000563 | Tyrosine hydroxylase | NP_001138794 | 0 |
|  | BGIBMGA012088 | Aspartate 1-decarboxylase | ABX89951 | 0 |
| NPLP | BGIBMGA011715 | Neuropeptide-like protein 4 | NP_001124370 | 0.13 |
|  | BGIBMGA011717 | Neuropeptide-like protein 4 | NP_001124370 | 0.16 |
|  | BGIBMGA011718 | Neuropeptide-like protein 4 | NP_001124370 | 0.0002 |
|  | BGIBMGA011762 | Neuropeptide-like protein 4 | NP_001124370 | 0.014 |
|  | BGIBMGA011763 | Neuropeptide-like protein 4 | NP_001124370 | 0.16 |
|  | BGIBMGA011764 | Neuropeptide-like protein 4 | NP_001124370 | 5.00E-26 |
| Others | BGIBMGA013242 | CG14439 | NP_572347 | 3.00E-174 |
|  | BGIBMGA011675 | Nuclear pore complex protein Nup160 homolog | XP_001121192 | 2.00E-59 |
|  | BGIBMGA012606 | Hypothetical protein SNOG_04660 | XP_001795073 | 0.002 |
|  | BGIBMGA005753 | Unknown | - | - |
|  | BGIBMGA002734 | Unknown | - | - |
|  | BGIBMGA001316 | Hypothetical protein Epulo_01906 | ZP_02691870 | 9.00E-07 |
|  | BGIBMGA000421 | AaeL_AAEL007483 | XP_001652778 | 3.00E-33 |
|  | BGIBMGA003530 | AaeL_AAEL005781 | XP_001651442 | 2.00E-09 |
|  | BGIBMGA002660 | Glycoside hydrolase | XP_001850321 | 1.00E-155 |
|  | BGIBMGA001052 | GI12087 | XP_002007969 | 9.00E-49 |
|  | BGIBMGA007322 | AGAP001907-PA | XP_321158 | 4.00E-28 |
|  | BGIBMGA006540 | Adenylate cyclase | XP_001659917 | 7.00E-180 |
|  |  |  |  |  |
| **Down-regulated genes** |  |  |  |  |
|  | BGIBMGA011266 | Sex-specific storage-protein 1 | NP_001106747 | 0 |
|  | BGIBMGA006874 | Chitinase-related protein 1 | NP_001036861 | 0 |

*BmorCPR124 **/** BmorCPR150: Cuticular protein genes BmorCPR124 and BmorCPR150 shared the same probe. JHBP represents Juvenile Hormone Binding Proteina and ERP represents Ecdysteroid Regulated Protein. NPLP represents Neuropeptide-Like Protein.
