# Supplementary material for: Expression profile of cuticular genes of silkworm, Bombyx mori
Source: BMC Genomics. 2010 Mar 15;11:173. doi: 10.1186/1471-2164-11-173 (PMC2848646; doi:10.1186/1471-2164-11-173)
Supplement: Additional file 3 — The cuticular protein genes in silkworm. It is a table that contains all the silkworm cuticular protein genes. The gene families belonging, gene symbol or names, and accession numbers are also given. [file 1471-2164-11-173-S3.DOC]

Table 1 Cuticular protein genes in *Bombyx mori*

| Gene family Symbol/Name | Accession No | Gene family | Symbol/Name | Accession No |
| --- | --- | --- | --- | --- |
| RR-1 BmorCPR1  BmorCPR2  BmorCPR3  BmorCPR4  BmorCPR5  BmorCPR6  BmorCPR7  BmorCPR8  BmorCPR9  BmorCPR10  BmorCPR11  BmorCPR12  BmorCPR13  BmorCPR14  BmorCPR15  BmorCPR16  BmorCPR17  BmorCPR18  BmorCPR19  BmorCPR20  BmorCPR21  BmorCPR23  BmorCPR24  BmorCPR25  BmorCPR26  BmorCPR27  BmorCPR28  BmorCPR29  BmorCPR30  BmorCPR31  BmorCPR32  BmorCPR33  BmorCPR34  BmorCPR35  BmorCPR36  BmorCPR37  BmorCPR38  BmorCPR39  BmorCPR40  BmorCPR41  BmorCPR42  BmorCPR43  BmorCPR44  BmorCPR45  BmorCPR46  BmorCPR47  BmorCPR48  BmorCPR49  BmorCPR50  BmorCPR51  BmorCPR52  BmorCPR53  BmorCPR54  BmorCPR55  BmorCPR56 | BR000502  BR000503  BR000504  BR000505  BR000506  BR000507  BR000508  BR000509  BR000510  BR000511  BR000512  BR000513  BR000514  BR000515  BR000516  BR000517  BR000518  BR000519  BR000520  BR000521  BR000522  BR000524  BR000525  BR000526  BR000527  BR000528  BR000529  BR000530＊  BR000531  BR000532  BR000533  BR000534  BR000535  BR000536  BR000537  BR000538  BR000539  BR000540  BR000541  BR000542  BR000543  BR000544  BR000545  BR000546  BR000547  BR000548  BR000549  BR000550  BR000551  BR000552  BR000553  BR000554  BR000555  BR000556  BR000557 | RR-2 | BmorCPR22  BmorCPR57  BmorCPR58  BmorCPR59  BmorCPR60  BmorCPR61  BmorCPR62  BmorCPR63  BmorCPR64  BmorCPR65  BmorCPR66  BmorCPR67  BmorCPR68  BmorCPR69  BmorCPR70  BmorCPR71  BmorCPR72  BmorCPR73  BmorCPR74  BmorCPR75  BmorCPR76  BmorCPR77  BmorCPR78  BmorCPR79  BmorCPR80  BmorCPR81  BmorCPR82  BmorCPR83  BmorCPR84  BmorCPR85  BmorCPR86  BmorCPR87  BmorCPR88  BmorCPR89  BmorCPR90  BmorCPR91  BmorCPR92  BmorCPR93  BmorCPR94  BmorCPR95  BmorCPR96  BmorCPR97  BmorCPR98  BmorCPR99  BmorCPR100  BmorCPR101  BmorCPR102  BmorCPR103  BmorCPR104  BmorCPR105  BmorCPR106  BmorCPR107  BmorCPR108  BmorCPR109  BmorCPR110 | BR000523▲  BR000558  BR000559  BR000560  BR000561  BR000562  BR000563  BR000564  BR000565  BR000566  BR000567＊  BR000568  BR000569  BR000570  BR000571  BR000572  BR000573  BR000574  BR000575  BR000576  BR000577  BR000578  BR000579  BR000580  BR000581  BR000582  BR000583  BR000584  BR000585  BR000586  BR000587  BR000588  BR000589  BR000590  BR000591  BR000592  BR000593  BR000594  BR000595  BR000596  BR000597  BR000598  BR000599  BR000600  BR000601  BR000602  BR000603^a^  BR000604^a^  BR000605  BR000606  BR000607  BR000608  BR000609  BR000610  BR000611 |

Table 1 continued

| Gene family Symbol/Name | Accession No | Gene family | Symbol/Name | Accession No |
| --- | --- | --- | --- | --- |
| RR-2 BmorCPR111  BmorCPR112  BmorCPR113  BmorCPR114  BmorCPR115  BmorCPR116  BmorCPR117  BmorCPR118  BmorCPR119  BmorCPR120  BmorCPR121  BmorCPR122  BmorCPR123  BmorCPR124  BmorCPR125  BmorCPR126  BmorCPR127  BmorCPR128  BmorCPR129  BmorCPR130  BmorCPR131  BmorCPR132  BmorCPR133  BmorCPR134  BmorCPR135  BmorCPR136  BmorCPR137  BmorCPR138  BmorCPR139  BmorCPR140  BmorCPR141  BmorCPR142  BmorCPR143  BmorCPR144  BmorCPR145  BmorCPR149  BmorCPR150  BmorCPR151  RR-3 BmorCPR146  BmorCPR147  BmorCPR148  Tweedle BmorCPT1  BmorCPT2  BmorCPT3  BmorCPT4  CPF BmorCPF  CPFL BmorCPFL1  BmorCPFL2  BmorCPFL3  BmorCPFL4  CPG BmorCPG1  BmorCPG2  BmorCPG3  BmorCPG4  BmorCPG5 | BR000612  BR000613  BR000614  BR000615  BR000616  BR000617  BR000618  BR000619  BR000620**^b^**  BR000621**^b^**  BR000622  BR000623  BR000624  BR000625**^c^**  BR000626  BR000627  BR000628  BR000629  BR000630  BR000631  BR000632  BR000633  BR000634  BR000635  BR000636  BR000637  BR000638  BR000639  BR000640  BR000641  BR000642  BR000643  BR000644  BR000645  BR000646  GU070696  GU070697**^c^**  GU070698  BR000647  BR000648  BR000649  BR000650  BR000651  BR000652  BR000653  BR000417  BR000418  BR000419  BR000420  BR000421  BR000422＊  BR000423  BR000424  BR000425  BR000426 | CPH | BmorCPG6  BmorCPG7  BmorCPG8  BmorCPG9  BmorCPG10  BmorCPG11  BmorCPG12  BmorCPG13  BmorCPG14  BmorCPG15  BmorCPG16  BmorCPG17  BmorCPG18  BmorCPG19  BmorCPG20  BmorCPG21  BmorCPG22  BmorCPG23  BmorCPG24  BmorCPG25  BmorCPG26  BmorCPG27  BmorCPG28  BmorCPG29  BmorCPG30  BmorCPG31  BmorCPG32  BmorCPG33  BmorCPG34  BmorCPG35  BmorCPG36  BmorCPG37  BmorCPG38  BmorCPG39  BmorCPG40  BmorCPG41  BmorCPG42  BmorCPG43  BmorCPG44  BmorCPG45  BmorCPG46  BmorCPG47  BmorCPG48  BmorCPG49  BmorCPG50  BmorCPG51  BmorCPH1  BmorCPH2  BmorCPH3  BmorCPH4  BmorCPH5  BmorCPH6  BmorCPH7  BmorCPH8  BmorCPH9 | BR000427  BR000428  BR000429  BR000430  BR000431  BR000432  BR000433  BR000434  BR000435  BR000436  BR000437  BR000438  BR000439＊  BR000440  BR000441  BR000442  BR000443  BR000444  BR000445  BR000446  BR000447**^d^**  BR000448**^d^**  BR000449**^d^**  BR000450＊  GU070699  GU070700  GU070701  GU070702  GU070703  GU070704  GU070705  GU070706  GU070707  GU070708  GU070709  GU070710  GU070711  GU070712  GU070713  GU070714  GU070715  GU070716  GU070717  GU070718  NM_001043496  GU070719  BR000451  BR000452  BR000453  BR000454  BR000457  BR000461  BR000462  BR000463  BR000464 |

Table 1 continued

| Gene family Symbol/Name | Accession No | Gene family | Symbol/Name | Accession No |
| --- | --- | --- | --- | --- |
| CPH BmorCPH10  BmorCPH11  BmorCPH12  BmorCPH13  BmorCPH14  BmorCPH15  BmorCPH16  BmorCPH17  BmorCPH18  BmorCPH19  BmorCPH20  BmorCPH21  BmorCPH22  BmorCPH23  BmorCPH24  BmorCPH25  BmorCPH26  BmorCPH27 | BR000465  BR000466  BR000467  BR000468  BR000469  BR000470  BR000471  BR000472  BR000473  BR000474  BR000475  BR000476  BR000477  BR000478  BR000486  BR000487  BR000488  BR000490 | CPH | BmorCPH28  BmorCPH29  BmorCPH30  BmorCPH31  BmorCPH32  BmorCPH33  BmorCPH34  BmorCPH35  BmorCPH36  BmorCPH37  BmorCPH38  BmorCPH39  BmorCPH40  BmorCPH41  BmorCPH42  BmorCPH43  BmorCPH44 | BR000493  BR000494＊  BR000495  BR000496  BR000497  BR000500  BR000501  GU070720**※**  GU070721**※**  GU070722**※**  GU070723**※**  GU070724**※**  GU070725**※**  GU070726**※**  GU070727**※**  GU070728**※**  GU070729**※** |

BmorCPR22 marked with “▲” was identified as RR-2 cuticular protein gene in this study, while it was previously identified as a RR-1 protein gene (Futahashi et al., 2008). No probes were matched for the genes marked with “＊”. Genes marked with letters a, b, c, and d shared probes. Ten CPH protein genes newly identified in this study were marked with “**※”.**
