# Supplementary material for: Expression profile of cuticular genes of silkworm, Bombyx mori
Source: BMC Genomics. 2010 Mar 15;11:173. doi: 10.1186/1471-2164-11-173 (PMC2848646; doi:10.1186/1471-2164-11-173)
Supplement: Additional file 4 — The alignment match logos of elements I and III with known transcription factors. This file contains two figures illustrating the alignment match logos of elements Iand III with known transcription factors. In each figure, the name of transcription factors and relative parameters are given. [file 1471-2164-11-173-S4.PPT]

## Slide 1
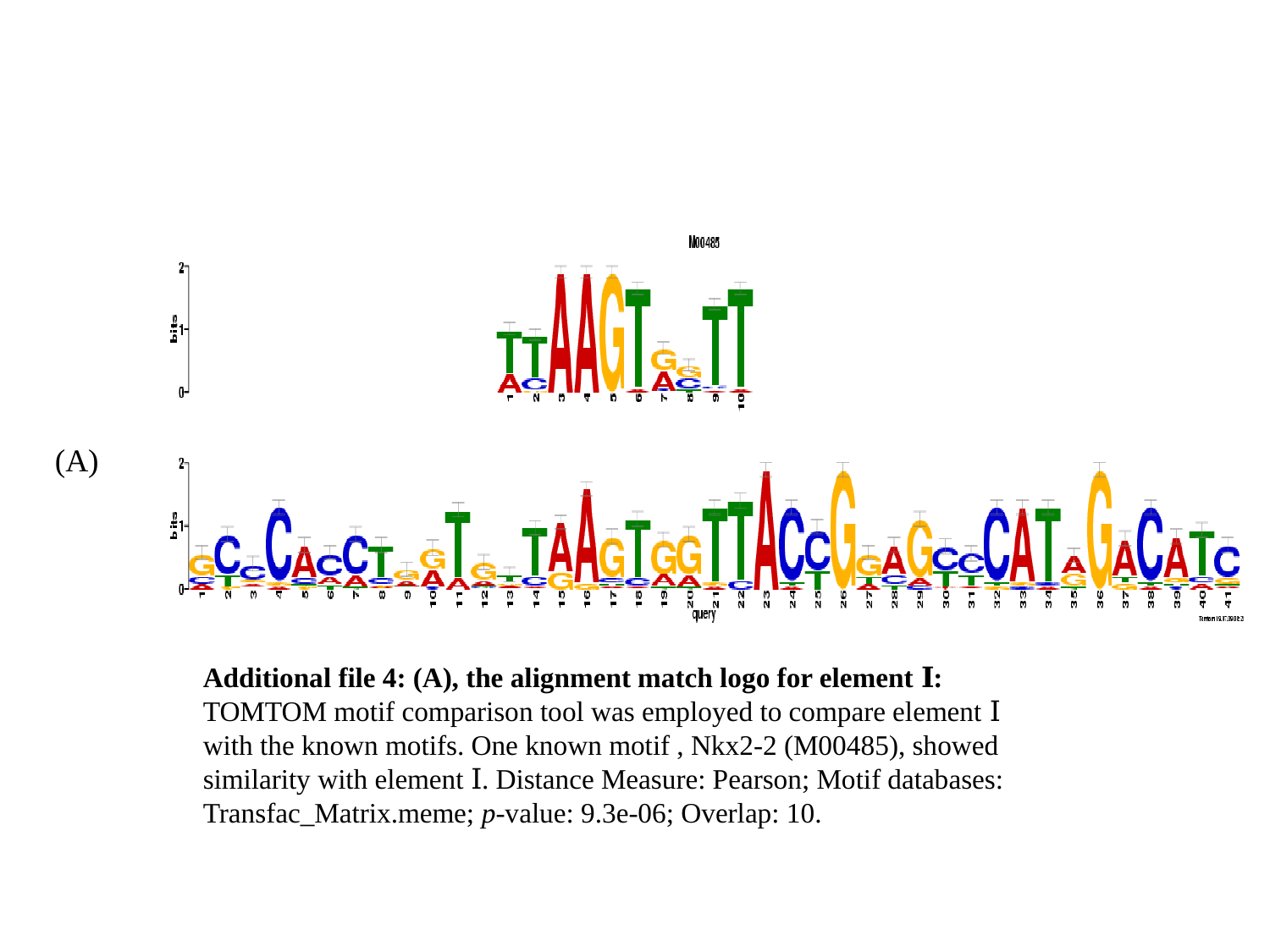

(A)
Additional file 4: (A), the alignment match logo for element Ⅰ:
TOMTOM motif comparison tool was employed to compare element Ⅰ with the known motifs. One known motif , Nkx2-2 (M00485), showed similarity with element Ⅰ. Distance Measure: Pearson; Motif databases: Transfac_Matrix.meme; p-value: 9.3e-06; Overlap: 10.

## Slide 2
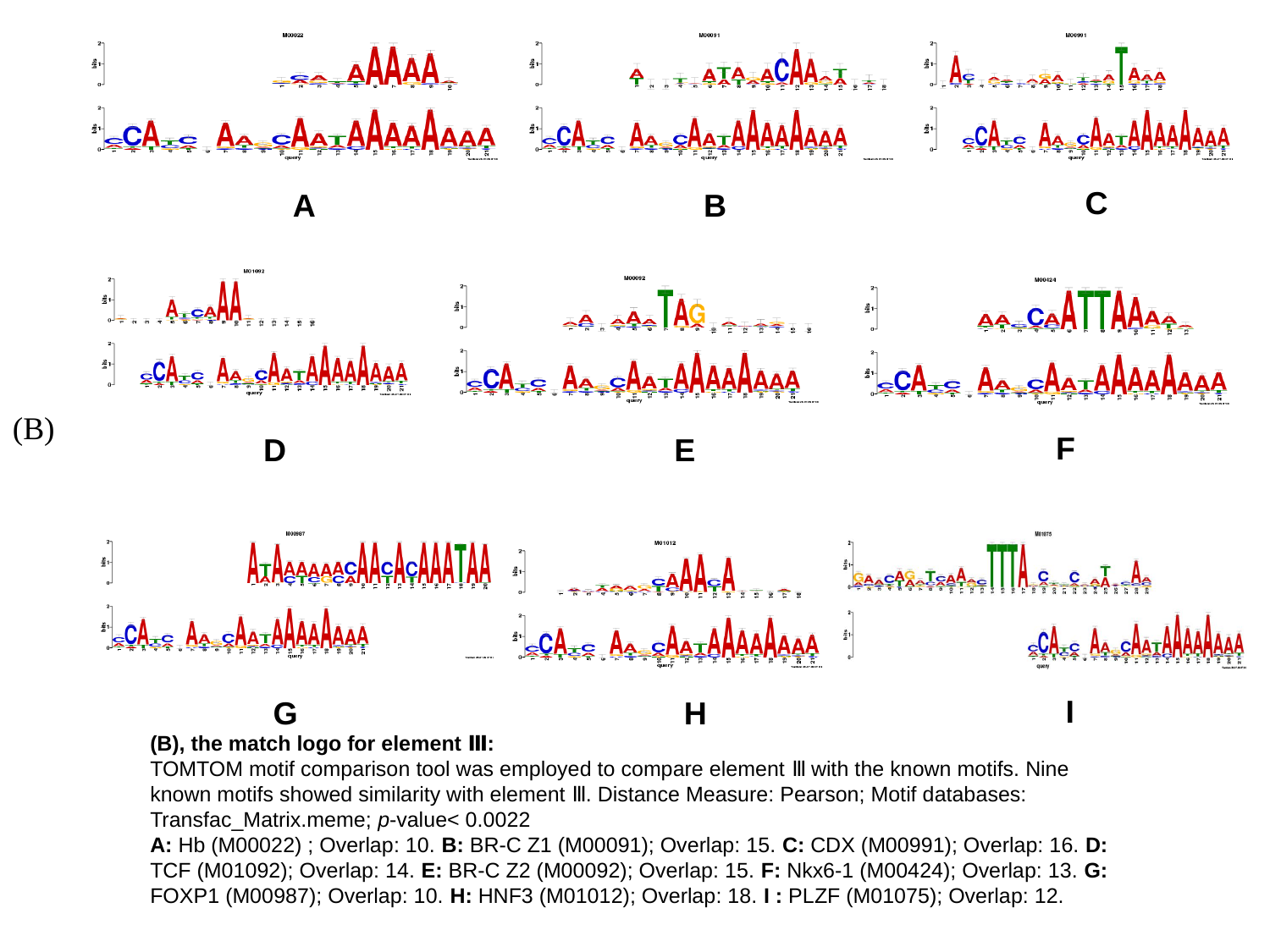

C
A
B
F
D
E
I
G
H
(B)
(B), the match logo for element Ⅲ:
TOMTOM motif comparison tool was employed to compare element Ⅲ with the known motifs. Nine known motifs showed similarity with element Ⅲ. Distance Measure: Pearson; Motif databases: Transfac_Matrix.meme; p-value< 0.0022
A: Hb (M00022) ; Overlap: 10. B: BR-C Z1 (M00091); Overlap: 15. C: CDX (M00991); Overlap: 16. D: TCF (M01092); Overlap: 14. E: BR-C Z2 (M00092); Overlap: 15. F: Nkx6-1 (M00424); Overlap: 13. G: FOXP1 (M00987); Overlap: 10. H: HNF3 (M01012); Overlap: 18. I : PLZF (M01075); Overlap: 12.
